# The Functional Relationship between NADPH Thioredoxin Reductase C, 2-Cys Peroxiredoxins, and *m*-Type Thioredoxins in the Regulation of Calvin–Benson Cycle and Malate-Valve Enzymes in Arabidopsis

**DOI:** 10.3390/antiox12051041

**Published:** 2023-05-03

**Authors:** Víctor Delgado-Requerey, Francisco Javier Cejudo, María-Cruz González

**Affiliations:** 1 Instituto de Bioquímica Vegetal y Fotosíntesis, Universidad de Sevilla and Consejo Superior de Investigaciones Científicas, Avenida Américo Vespucio 49, 41092 Sevilla, Spain; vicdelreq@gmail.com; 2 Departamento de Bioquímica Vegetal y Biología Molecular, Facultad de Biología, Universidad de Sevilla, 41012 Sevilla, Spain

**Keywords:** Calvin–Benson cycle, chloroplast, 2-Cys peroxiredoxin, malate valve, NADPH-dependent Trx reductase (NTRC), redox regulation, thioredoxin

## Abstract

The concerted regulation of chloroplast biosynthetic pathways and NADPH extrusion via malate valve depends on *f* and *m* thioredoxins (Trxs). The finding that decreased levels of the thiol-peroxidase 2-Cys peroxiredoxin (Prx) suppress the severe phenotype of Arabidopsis mutants lacking NADPH-dependent Trx reductase C (NTRC) and Trxs *f* uncovered the central function of the NTRC-2-Cys-Prx redox system in chloroplast performance. These results suggest that Trxs *m* are also regulated by this system; however, the functional relationship between NTRC, 2-Cys Prxs, and *m-*type Trxs is unknown. To address this issue, we generated *Arabidopsis thaliana* mutants combining deficiencies in NTRC, 2-Cys Prx B, Trxs *m*1, and *m*4. The single *trxm1* and *trxm4* mutants showed a wild-type phenotype, growth retardation being noticed only in the *trxm1m4* double mutant. Moreover, the *ntrc-trxm1m4* mutant displayed a more severe phenotype than the *ntrc* mutant, as shown by the impaired photosynthetic performance, altered chloroplast structure, and defective light-dependent reduction in the Calvin–Benson cycle and malate-valve enzymes. These effects were suppressed by the decreased contents of 2-Cys Prx, since the quadruple *ntrc-trxm1m4-2cpb* mutant displayed a wild-type-like phenotype. These results show that the activity of *m*-type Trxs in the light-dependent regulation of biosynthetic enzymes and malate valve is controlled by the NTRC-2-Cys-Prx system.

## 1. Introduction

Photosynthesis is the process that generates biomass and oxygen in the biosphere; thus, it is essential for life on Earth. This process adjusts to the growth conditions of photosynthetic organisms, from algae, such as the streptophyte class Zygnematophyecae, to plants [1,2]. As sessile organisms, plants face different and continuous changes in their environmental conditions, so the rapid adaptation of their photosynthetic performance to the environment is essential for their survival and productivity [3]. One of the main mechanisms controlling the metabolic state of chloroplasts in response to environmental cues is based on the dithiol–disulphide exchange of usually well-conserved cysteine residues of redox-regulated enzymes, in which the disulphide-reductase activity of thioredoxins (Trxs) plays a key role. Trxs are small proteins, 12–14 kDa, which were first discovered in bacteria but are present in all types of organisms [4,5]. Their active site is composed of a dithiol, CXXC, which is essential for disulphide-reduction activity. The two Cys in the active site are separated by two amino acids (Gly/Pro, Pro), which are necessary to maintain the conformation of the active site, forming the canonical WCG/PPC active site of typical Trxs [6]. Plants harbour a large gene family encoding different isoforms of Trxs, which are present in all subcellular compartments [6], but these enzymes are especially abundant in the chloroplast, reflecting the paramount importance of Trx-dependent redox regulation for photosynthetic performance [7,8,9].

Reduction of Trx in non-photosynthetic tissues relies on NADPH as a source of reducing power, which is catalysed by NADPH-dependent thioredoxin reductase (NTR) [10]. In contrast, two different redox systems have been described in chloroplasts. Reduced ferredoxin (Fd) generated by the light-driven photosynthetic electron-transport chain is the major source of reducing power for chloroplast Trxs, in a process mediated by a Fd-dependent thioredoxin reductase (FTR) [11]. It is well established that the Fd–FTR–Trxs redox system is responsible for the reductive activation of enzymes in the Calvin–Benson cycle (CBC) during the day [11,12]. Oxygenic photosynthetic organisms possess an additional system, termed NTRC, an NTR with a joint Trx domain at the C-terminus [13], which efficiently reduces the antioxidant enzyme 2-Cys peroxiredoxin (2-Cys Prx) [14,15,16]. Since NTRC shows a high affinity for NADPH [17], this enzyme is also functional during the night, when NADPH can be generated by the oxidative pentose phosphate pathway (OPPP) [15,18].

Several studies have addressed the functional relationship of the Fd–FTR–Trxs and NTRC redox systems in the regulation of chloroplast metabolism. The characterization of the Arabidopsis knockout mutant devoid of NTRC showed the participation of this enzyme in the regulation of metabolic pathways that were previously known to be regulated by Trxs, such as the biosynthesis of starch [19,20] and chlorophyll [21,22,23], suggesting that the Fd–FTR–Trxs and NTRC redox systems overlap to some extent. Although the list of putative targets of NTRC so far identified [24,25,26] is much shorter than that of the putative targets of Trxs [27], most of the targets are common to both systems. Furthermore, the lethal phenotype of Arabidopsis mutants simultaneously devoid of the catalytic subunit of FTR and NTRC, only partially rescued under heterotrophic conditions, suggests that both systems act cooperatively and are essential for plant growth [24]. However, single mutants defective in the catalytic subunit of FTR (FTRc) and NTRC show different growth phenotypes, suggesting specific functions for each system [24]. In this regard, it was proposed that NTRC plays a relevant role in photosynthesis regulation in dark–light and low–high light transitions, when the activity of the Fd–FTR–Trx system is limited [28]. In addition, NTRC has been shown to be an indispensable regulator of photosynthesis in young leaves [29].

Arabidopsis chloroplasts are equipped with a complex set of Trxs, including both atypical and typical isoforms, such as those of the types *m*, *f*, *x*, *y*, and *z* [6]. The highly abundant *m*- and *f*-type Trxs are mainly involved in the regulation of CBC enzymes, including fructose-1,6-bisphosphatase (FBPase), sedoheptulose-1,7-bisphosphatase (SBPase), and the malate-valve NADP-malate dehydrogenase (NADP-MDH) [24,30,31,32,33]. The less abundant Trxs, of the types *x* and *y*, were proposed to be relevant in antioxidant defence, based on their activity as reductants of Prxs [30,31]. Finally, Trx *z* participates in the redox regulation of plastid-encoded RNA polymerase (PEP)-dependent gene expression [34]. Since Trx *z* interacts with FTRc, the participation of FTR in the regulation of plastid gene expression in the early stages of plant development has been suggested [35].

Additional analyses of the functional relationship between the Fd–FTR–Trx and NTRC redox systems was addressed by the generation of multiple mutants. The severe growth-inhibition phenotype of Arabidopsis mutants lacking NTRC and Trxs *f* or *x* [36,37,38] suggested the concerted action of the Fd–FTR–Trxs and NTRC systems, which play a relevant role in early plant development and in chloroplast biogenesis. However, the combined deficiency of NTRC and the Trxs *y*1 and *y*2 resulted only in slight growth retardation compared to *ntrc* plants, and had no effect on photosynthetic performance or the light-dependent reduction in CBC enzymes [39]. On the other hand, the finding that decreased levels of 2-Cys Prxs suppress the phenotype caused by the deficiencies of NTRC and NTRC plus Trxs *f* or *x* [40,41] led to the proposal that the redox balance of 2-Cys Prxs integrates the activities of the Fd–FTR–Trxs and NTRC redox systems. 

Altogether, the approaches based on the combination of the *ntrc* mutant with mutants lacking Trxs of the types *f*, *x*, and *y* suggest that the NTRC-2-Cys-Prxs system controls the activity of any plastid Trx. However, the genetic relationship between NTRC and Trxs *m* has been poorly analysed. The Arabidopsis chloroplast harbours four isoforms of *m*-type Trxs. Of these, Trx *m*1, *m*2, and *m*4 are more abundant than the *m*3 isoform [32], which was proposed to participate in plasmodesmata communication [42]. The virus-induced gene silencing (VIGS) of the *Trx m1, Trx m2*, and *Trx m4* genes in a *ntrc* background resulted in decreased chlorophyll content and retarded growth compared to *ntrc* plants and decreased Mg protoporphyrin methyl transferase (CHLM) activity, showing that both redox systems cooperatively regulate CHLM and tetrapyrrole biosynthesis [43]. However, the effect of simultaneous deficiencies in NTRC and the specific *m*-type Trxs on other aspects of plant development and the regulation of chloroplast metabolism has not been analysed so far. Similarly, it is currently unknown whether 2-Cys Prxs exert any effect on the activity of Trxs *m*. Thus, with the aim of obtaining a deeper insight into the functional relationship between Trxs *m*, NTRC, and 2-Cys Prxs, we generated mutant plants simultaneously devoid of NTRC, Trxs *m*1 and *m*4, and 2-Cys Prx B. With the aid of these mutants, we analysed the concerted light-dependent regulation of enzymes involved in chloroplast biosynthetic pathways and NADPH extrusion via the malate valve. Our results lend support to the notion that 2-Cys Prxs play a central function in chloroplast-redox regulation.

## 2. Materials and Methods

### 2.1. Biological Materials and Growth Conditions

*Arabidopsis thaliana* wild-type (WT, ecotype Columbia) and mutant plants were grown in soil in growth chambers under long-day (LD, 16-h light/8-h darkness) or short-day (SD, 8-h light/16-h darkness) photoperiods, with a light intensity of 120 μE m^−2^ s^−1^, at temperatures of 22 °C in light and 20 °C in darkness, respectively. The single *trxm1* (SALK_087118) and *trxm4* (SALK_032538) mutants [44,45] were manually crossed to generate double *trxm1m4* mutant. To obtain the combination of these mutants and *ntrc*, the *ntrc* mutant [13] was manually crossed with both the *trxm1* and the *trxm4* mutants to generate the *ntrc-trxm1* and *ntrc-trxm4* double mutants, which were subsequently crossed to generate the *ntrc-trxm1m4* triple mutant. The quadruple *ntrc-trxm1m4-2cpb* mutant was obtained by crossing the triple *ntrc-trxm1m4* mutant with the Δ*2cp* mutant [46]. Seeds from each cross were checked for heterozygosity of the T-DNA insertions in the corresponding genes. Plants were then allowed to self-pollinize, and the homozygous plants identified in the progeny by PCR analysis of genomic DNA with the oligonucleotides listed in Table S1. *Escherichia coli* and *Agrobacterium tumefaciens* were grown in liquid Luria-Bertani nutrient medium (LB) [47] at 37 °C and 28 °C, respectively, with the appropriate antibiotics.

### 2.2. RNA Extraction, cDNA Synthesis, and Quantitative RT-PCR Analysis

Total RNA was extracted from 100 mg of liquid-nitrogen-frozen material using TRIzol reagent (Invitrogen). The cDNA was synthesized from 1.5 μg of total RNA using the Maxima first-strand cDNA synthesis kit (Fermentas), following the manufacturer’s instructions. The qPCR analysis was performed using the iQ5 Multicolor Real-Time PCR Detection System (Bio-Rad), with a standard amplification protocol (95 °C, 3 min; 40 cycles of 95 °C, 10 s; 60 °C, 30 s), utilising the oligonucleotides listed in Table S2. Oligonucleotide-hybridisation specificity and non-specific amplification were checked after the PCR, based on the analysis of a melting curve (55–94 °C at 0.5 °C per 30 s). Expression levels were referred to the levels of *UBIQUITIN* and *ACTIN* transcripts in the corresponding sample.

### 2.3. Protein Extraction, Alkylation Assays, and Western-Blot Analysis

For the extraction of proteins, approximately 200 mg of ground frozen material was homogenized in protein-extraction buffer (100 mM Tris-HCl, pH 7.9, 10 mM MgCl_2_, 1 mM EDTA, 1 mM PMSF, 10% (*v*/*v*) glycerol, 1% (*v*/*v*) protease inhibitor cocktail (Sigma-Aldrich)). Proteins were quantified with the Bradford reagent, subjected to reducing or non-reducing SDS-PAGE, using the acrylamide concentrations specified in the figure legends, and electro-blotted onto nitrocellulose membranes. For the analysis of the redox state, proteins were extracted in 10% (*v*/*v*) trichloroacetic acid (350 µL TCA/100 mg of tissue) to quench thiol oxidation, as previously described [33]. Once centrifuged, proteins in the pellet were resuspended in alkylation buffer (2% SDS, 50 mM Tris-HCl, pH 7.8, 2.5% (*v*/*v*) glycerol, 4 M urea) containing 10 mM methyl-maleimide polyethylene glycol (MM-PEG_24_) and incubated for 20 min at room temperature. After electrophoresis, proteins were transferred to nitrocellulose membranes. Anti-NTRC- and anti-2-Cys-Prx-specific antibodies were previously raised in our laboratory [13,15]. Anti-FBPase and anti-Trx *m* antibodies were kindly provided by Dr. Sahrawy (Estación Experimental del Zaidín, Granada, Spain) and Dr. Buchanan (Department of Plant and Microbial Biology, University of California, Berkeley, CA, USA), respectively. Anti-PRK antibody was purchased from Agrisera. A specific anti-NADP-MDH antibody was raised at the Servicio de Producción Animal (University of Seville, Seville, Spain) by rabbit immunization with purified recombinant Arabidopsis NADP-MDH.

### 2.4. Analysis of Chlorophyll Content and Measurements of Chlorophyll a Fluorescence

For pigment extraction, leaf discs were weighed and incubated in 1 mL methanol overnight at 4 °C. Once extracted, chlorophyll concentration was determined spectrophotometrically, as described in [48], and normalized to fresh weight or disc area. The values were compared with a Tukey test (Anova) using a confidence interval of 95%.

The PSII chlorophyll *a* fluorescence was measured using an Imaging Pam M-series chlorophyll fluorescence system (DUAL-PAM-100; Walz, Effeltrich, Germany). For the analysis of PSII maximum quantum yield, Fv/Fm, plants were dark-adapted for 30 min and a single saturating light pulse (λ = 635 nm) at 10,000 μE m^−2^ s^−1^ was applied. Induction-recovery curves were created using actinic light (λ = 635 nm) at 80 μE m^−2^ s^−1^ for 8 min. Every 60 s, a saturating pulse of actinic light at 10,000 μE m^−2^ s^−1^ and 0.60 s was applied and recovery in darkness was recorded for up to 6 min. The Y(II) and Y(NPQ) parameters, which corresponded to the quantum yields of PSII photochemistry and non-photochemical quenching (NPQ), respectively, were calculated using the ImagingWinGigE software according to the equations reported in [49]. Relative linear electron-transport rates, ETR(II), were measured in leaves of pre-illuminated plants by applying stepwise increasing actinic light intensities up to 1250 μE m^−2^ s^−1^.

### 2.5. Expression of NADP-MDH, Trxs m1, m2, and m4 in E. coli, Purification of the Recombinant Proteins, and In Vitro Activity Assays

Plasmids were designed for the expression of N-terminal His-tagged recombinant proteins. To this end, the coding sequences of Arabidopsis Trxs *m*1, *m*2, *m*4, and NADP-MDH, excluding the chloroplast-transit peptides, were amplified by PCR, using the oligonucleotides included in Table S3, and cloned into the pQE30-expression vector. Plasmids for Arabidopsis Trx *f*2 [38] and Trx *y*2 [39] were previously reported. The recombinant N-terminal His-tagged NTRC from rice, previously obtained [15], was used as source of the enzyme for in vitro assays. All recombinant proteins were expressed in XL1-Blue cells and purified by affinity chromatography using Nickel columns, as previously reported [15] (Figure S2).

In vitro assays of the reduction of NADP-MDH by NTRC and the different Trxs were performed by pre-incubation of 2 μM NADP-MDH with 1 μM of either NTRC, in the presence of 250 μM NADPH, or the corresponding Trx, in the presence of 500 μM DTT, for 30 min. For the determination of NADP-MDH activity, half of the sample was added to a reaction mix (10 mM sodium phosphate, pH 7.4) containing 300 μM NADPH and 300 μM oxaloacetate, with NADP-MDH at a final concentration of 50 nM. The rate of NADPH oxidation was measured in a Varioskan Lux multiple-well plate reader (Thermo Scientific, Tokyo, Japan) for 10 min. The NADP-MDH activity was determined as nmol of NADPH consumed per minute and µg of NADP-MDH. Enzyme-activity data were analysed for statistical significance with one-way ANOVA, followed by Tukey’s multiple-comparison test with a 95% confidence interval.

## 3. Results

### 3.1. The Combined Deficiency in NTRC and Trxs m Aggravates the Growth-Inhibition Phenotype Caused by the Deficiency in NTRC

**Figure 1:**
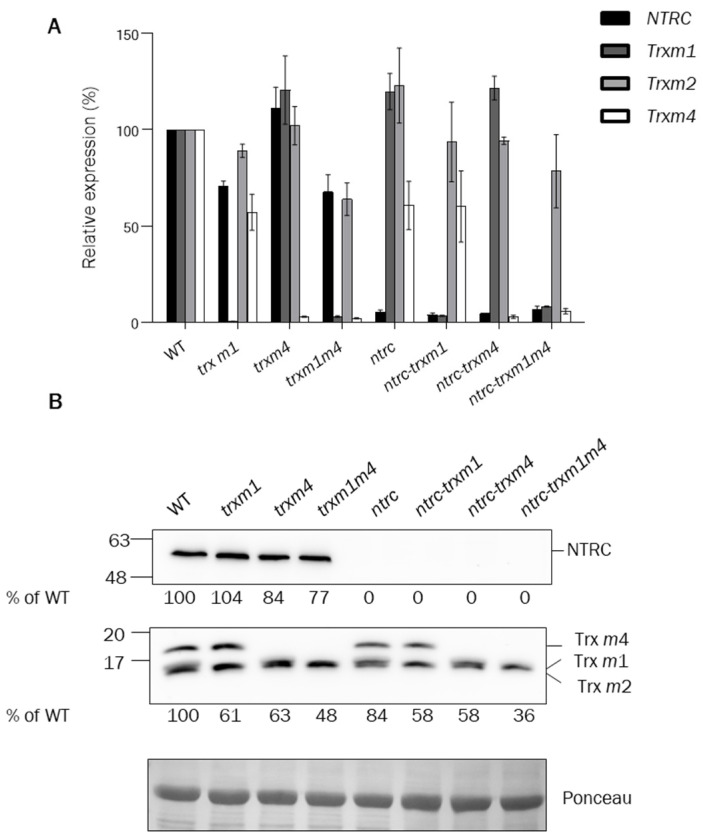
**Analysis of *NTRC*, *Trx m1*, *Trx m2*, and *Trx m4* expression and protein content in wild-type and mutant plants.** (**A**) The levels of expression of *Trx m1*, *Trx m2*, *Trx m4*, and *NTRC* in the wild-type and mutant plants was determined by RT-qPCR with total RNA extracted from rosette leaves. The oligonucleotides used for cDNA amplification are indicated in Table S2. Transcript levels were normalized to *UBIQUITIN* and *ACTIN* amplification and referred to the levels of expression in wild-type plants. Determinations were performed three times and mean values ± SE are represented. The content of NTRC and/or Trxs *m* (**B**) in wild-type and mutant lines was analysed by Western blot. Proteins were extracted from leaves of plants grown under long-day conditions for 3 weeks and subjected to SDS-PAGE (14% polyacrylamide) under reducing conditions, transferred to a nitrocellulose membrane, and probed with an anti-NTRC or anti-Trx *m* antibody. Ponceau staining was used as a loading control. The amount of NTRC and Trxs *m* in the different lines was quantified and referred to the amount of protein in the Ponceau staining. A representative blot is shown.

The first aim of this work was to establish the genetic interaction between NTRC and Trxs *m*, of which Arabidopsis contains four isoforms. Trx *m*3 was not included in this analysis based on the low level of expression of this gene and the chloroplast-unrelated function of this isoform in meristem maintenance [42]. Previous analyses showed that available *trxm2* mutants contain reduced levels of the corresponding transcript and protein, which are further increased in double and triple mutants [44,45,50]. Consequently, we focused on Trxs *m*1 and *m*4, from which knockout mutants were obtained, to establish their genetic interaction with NTRC. To this end, we generated the following combinations of mutants: *trxm1m4*, *ntrc-trxm1,* and *ntrc-trxm4* double mutants, and the *ntrc-trxm1m4* triple mutant. The RT-qPCR analyses showed the lack of transcripts of the corresponding genes in each of the lines (Figure 1A). The levels of Trx *m* proteins were analysed with an anti-Trx *m* antibody raised against purified Trx *m* from spinach, which effectively recognised Trxs *m*1, *m*2, and *m*4 from Arabidopsis (Figure 1B). In addition, the anti-NTRC antibody confirmed the absence of NTRC in the *ntrc* mutant lines (Figure 1B). Therefore, these results show that the single and multiple mutants generated in this study are knockouts for NTRC, Trx *m*1, and Trx *m*4. So far, knockout mutants simultaneously devoid of Trxs *m*1, *m2* and *m*4, have not been obtained. In addition, the silencing of these three Trxs *m* isoforms by VIGS and RNAi provoked seedling lethality due to the strong decrease in PSII activity, showing a dose-dependent effect between Trxs *m* levels and plant performance [32,50]. Therefore, the generated *ntrc-trxm1m4* plants were considered appropriate tools for analysing the genetic interaction between NTRC and *m*-type Trxs.

**Figure 2:**
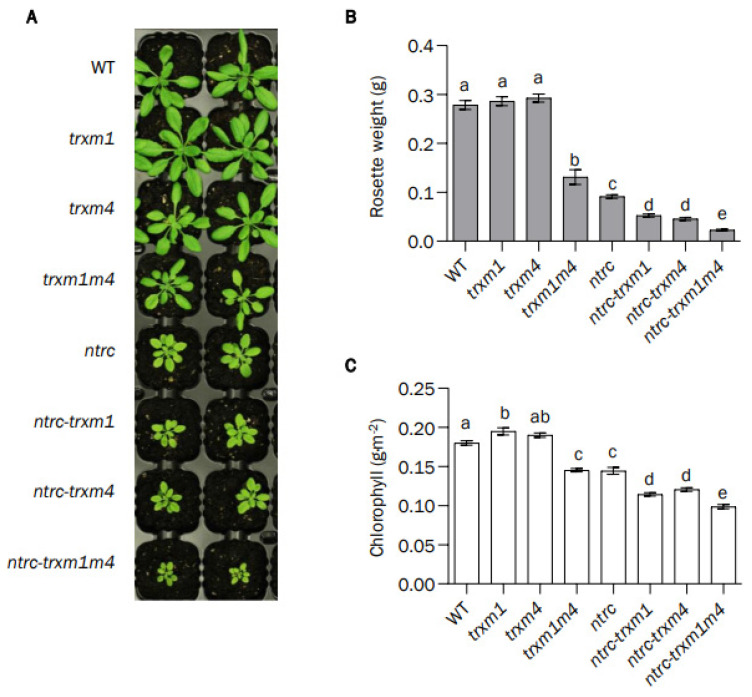
**Phenotypic characterization of plants lacking NTRC or/and Trxs *m*.** (**A**) Phenotype of wild-type and mutant plants grown under long-day conditions at 120 μE m^−2^ s^−1^ during 22 days. (**B**) The weights of rosette leaves from at least ten plants are presented as average values ± SE. (**C**) Chlorophyll content was determined from leaf discs (*n* = 10), and average values ± SE are represented. Letters indicate significant differences (*p* < 0.05) determined by one-way ANOVA, followed by Tukey’s test.

In agreement with previous results [32,51], the single *trxm1* and *trxm4* mutants showed growth phenotypes indistinguishable from that of the wild type when grown under long-day conditions, as shown by the similar rosette fresh weight (Figure 2A,B), although these lines showed slightly higher contents of chlorophyll (Figure 2C). However, the *trxm1m4* double mutant showed significant growth inhibition compared with the wild type and the single mutants (Figure 2A,B), confirming the relevance of these Trxs to plant growth. The combined deficiency of either Trx *m*1 or Trx *m*4 and NTRC resulted in an additional aggravation of the growth-inhibition phenotype of the *ntrc* mutant, which was even more severe in the case of the *ntrc-trxm1m4* mutant, in terms of both the rosette fresh weight (Figure 2A,B) and the chlorophyll content (Figure 2C). Thus, the severe consequences for plant growth of the simultaneous deficiency in NTRC and the Trxs *m*1 and *m*4 indicates that NTRC activity is required for the function of these Trxs.

### 3.2. The Simultaneous Deficiency in Trxs m1 and m4 and NTRC Has an Additive Effect on Photosynthetic Performance and Chloroplast Ultrastructure

**Figure 3:**
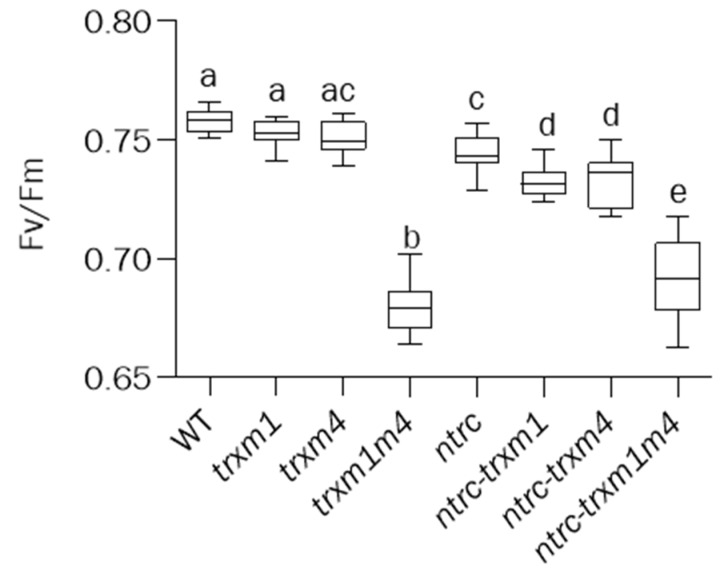
**Effects of the combined deficiencies of NTRC or/and Trxs *m* on Fv/Fm.** The maximum PSII quantum yield was determined as variable fluorescence (Fv) to maximal fluorescence (Fm), Fv/Fm, in dark-adapted leaves of plants grown under long-day conditions for 22 days. Box plots indicating the Fv/Fm value are shown. In each case, the median (segment inside rectangle), upper and lower quartiles (boxes), and minimum and maximum values (whiskers) are indicated. Letters indicate significant differences (*p* < 0.05), determined by one-way ANOVA, followed by Tukey’s post-test.

Once the concerted effects of the NTRC and Trxs *m*1 and *m*4 on the plant growth was established, we set out to investigate the effect of these deficiencies on the photosynthetic machinery. To this end, chlorophyll-fluorescence measurements were used to determine the efficiency of the light-energy utilization by the different mutants under analysis. The Fv/Fm ratio of the variable-to-maximal fluorescence in the dark-adapted leaves, which was a measure of the integrity of the PSII, was affected by the simultaneous deficiency in Trxs *m*1 and *m*4, both in the wild-type and the *ntrc* background, whereas the rest of the lines analysed showed only minor variations (Figure 3), indicating the relevant role of these Trxs in PSII stability, which seems to be independent of NTRC.

**Figure 4:**
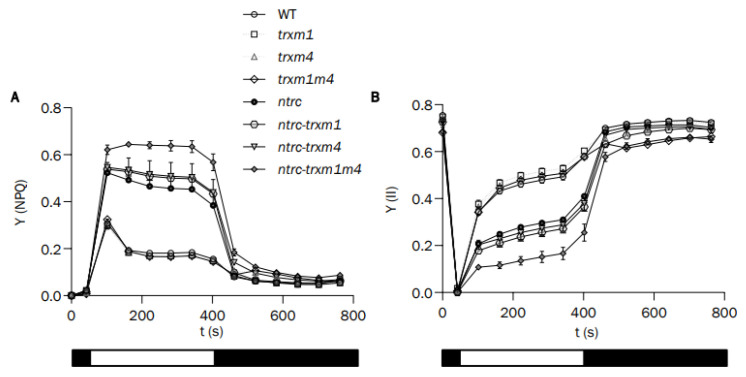
**Effects of NTRC and Trxs *m* deficiency on photosynthetic performance**. Photosynthetic parameters were measured on whole rosettes of the wild-type and mutant plants, as indicated, grown for 22 days under long-day conditions. (**A**) Non-photochemical quenching, Y(NPQ), and (**B**) quantum yields of photosystem II photochemistry, Y(II). Each value is the average of four determinations, and mean values ± SE are represented. White and black blocks indicate periods of illumination with actinic light (81 μE m^−2^ s^−1^) and darkness, respectively.

Non-photochemical quenching (NPQ) is a measurement of the thermal-energy dissipation when an excess of light is absorbed by PSII antenna, which acts as a compensatory mechanism to prevent PSII damage at low and medium light intensities. When the dark-adapted leaves were subjected to actinic light, a transient NPQ peak was observed, which was due to the acidification of the thylakoid lumen before photosynthesis was activated. While the single *trxm1* and *trxm4* and the double *trxm1m4* showed NPQ values indistinguishable from those of the wild type (Figure 4A), all the combinations in the *ntrc* background showed higher NPQ values, which were even higher in the case of the *ntrc-trxm1m4* mutant (Figure 4A). Accordingly, lower Y(II) values were determined in the NTRC-deficient plants, particularly in the *ntrc-trxm1m4* triple mutant (Figure 4B).

**Figure 5:**
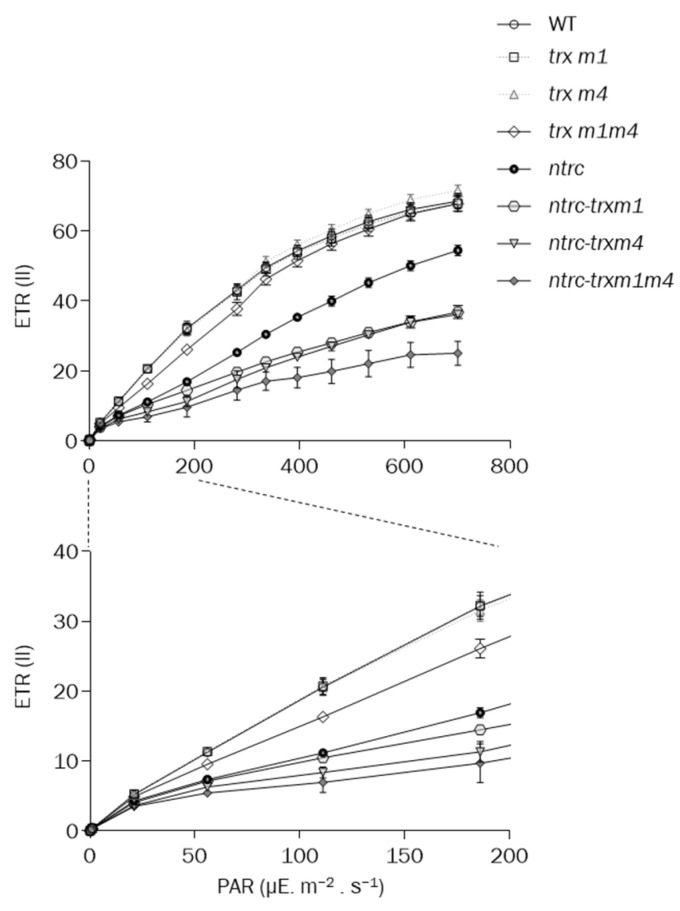
**Linear photosynthetic ETR in wildtype and mutant plants**. Relative ETRs of PSII, ETR(II), were determined during stepwise increase in photosynthetically active radiation (PAR) in plants grown as in Figure 4. The ETR(II) was determined in 3 replicates, and each data point is the mean ± SE. A closer representation of this parameter’s values measured in growth-like conditions is shown in the lower image.

Finally, the relative linear electron-transport rate of the PSII, ETR(II), was highly decreased in the triple *ntrc-trxm1m4* mutants under all the conditions tested, whereas the *ntrc-trxm1* and *ntrc-trxm4* plants showed lower ETR(II) values than the *ntrc* mutant (Figure 5). Although no differences in Y(II) and NPQ were observed for the *trxm1m4* mutant, this mutant showed lower values of ETR(II) than the wild type and the single *trxm1* and *trxm4* mutants at low light intensities (Figure 5).

**Figure 6:**
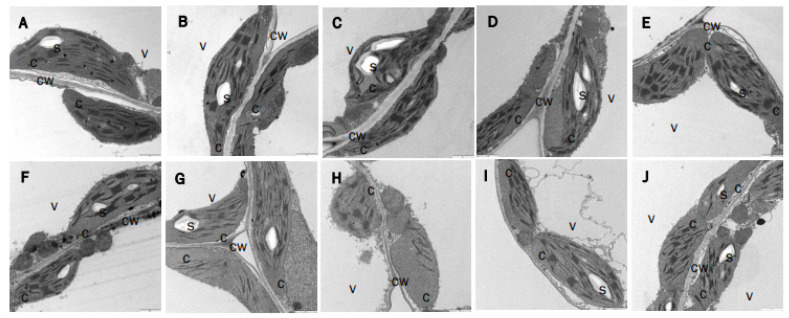
**Transmission-electron-microscopy analysis of chloroplast structure of wild-type and mutant plants.** Leaves of WT (**A**), *trxm1* (**B**), *trxm4* (**C**), *trxm1m4* (**D**), *ntrc* (**E**), *ntrc-trxm1* (**F**), *ntrc-trxm4* (**G**), and *ntrc-trxm1m4* (**H**–**J**) plants cultured under long-day photoperiod were collected after 25 days. c, chloroplasts; cw, cell wall; s, starch granules; v, vacuoles. Bars = 1 µm.

As an additional approach to determining the effects of NTRC and the Trxs *m*1 and *m*4 on the photosynthetic machinery, we analysed the chloroplast structure in the lines under study (Figure 6). The chloroplasts of the single mutants *trxm1* (Figure 6B) and *trxm4* (Figure 6C) were indistinguishable from those in the wild type (Figure 6A). In contrast, the *ntrc-trxm1m4* mutant showed greater heterogeneity in its chloroplast morphology, and more severe alterations of the thylakoid membrane network (Figure 6H–J), than the *ntrc* (Figure 6E) and the double mutants *trxm1m4* (Figure 6D), *ntrc-trxm1* (Figure 6F) and *ntrc-trxm4* (Figure 6G), which agreed with the more severe growth phenotype and the impairment of photosynthetic performance of the *ntrc-trxm1m4* mutant. Thus, the additive effect of the lack of Trxs *m*1 and *m*4 and of NTRC resulted in an increased number of chloroplasts with poor contents of thylakoid membranes and severe symptoms of chloroplast-structure alteration.

### 3.3. The Function of Trxs m1 and m4 in the Light-Dependent Regulation of Calvin–Benson Cycle and Malate-Valve Enzymes Requires NTRC Activity

**Figure 7:**
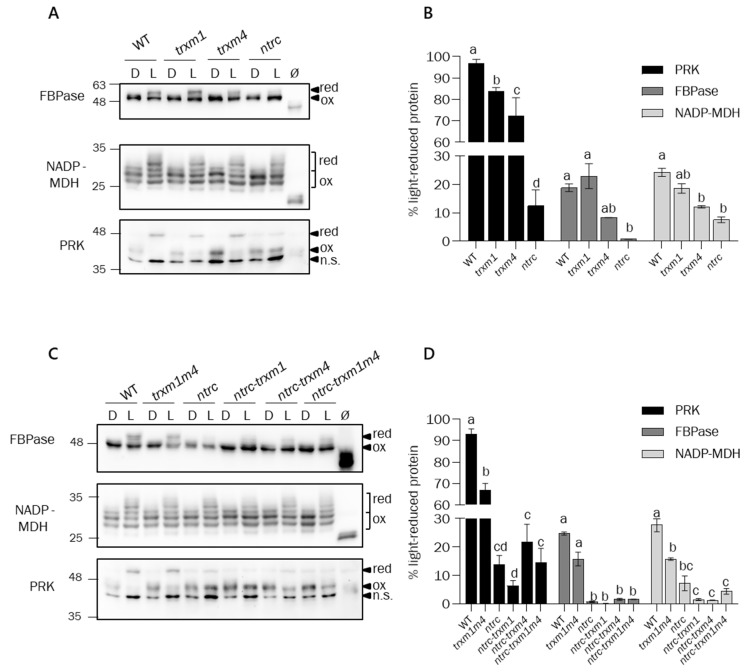
**Analysis of the in vivo redox state of chloroplast proteins in plants lacking NTRC and/or Trxs *m.*** Wild-type and mutant plants were grown under long-day conditions for 22 days at a light intensity of 120 µE m^−2^ s^−1^. Plants were incubated in darkness for 5 h and dark-adapted samples were collected (D). Subsequently, light was switched on and samples were taken after 30 min at a light intensity of 480 µE m^−2^ s^−1^ (L). The in vivo redox states of FBPase, NADP-MDH, and PRK were determined in WT, single-mutant plants (**A**,**B**), and in multiple mutants (**C**,**D**). Proteins were extracted in the presence of 10% TCA to preserve the thiol-redox state. Protein thiols were alkylated with 10 mM MM(PEG)_24_ prior to electrophoresis in a 9.5% polyacrylamide gel under reducing conditions, transferred to a nitrocellulose membrane, and probed with the indicated antibodies. Red, reduced; ox, oxidized; n.s., non-specific band; *Ø;* non-alkylated sample. (**B**,**D**) The corresponding band intensities were quantified (GelAnalyzer), and the percentage of reduction is the ratio between the reduced form and the sum of reduced and oxidized forms for each protein. Each value is the mean of three independent experiments ± SE. Letters indicate significant differences (*p* < 0.05), determined by one-way ANOVA, followed by Tukey’s post-test.

A key function of the chloroplast-redox regulatory network is the adjustment of metabolic biosynthetic pathways, such as carbon fixation via the CBC, in response to changes in light intensity. This adjustment needs to be coordinated with mechanisms for releasing excess reducing equivalents via the malate valve [52]. Thus, with the aim of establishing the function of NTRC and Trxs *m*1 and *m*4 in the light-dependent regulation of these processes, we analysed the in vivo redox state of well-known redox-regulated enzymes in the CBC, such as FBPase and PRK, and NADP-MDH, which is responsible for the malate valve. To this end, plants of the lines under analysis were subjected to dark-to-light (30 min at 480 μE m^−2^ s^−1^) transitions, and the redox state of the corresponding enzyme was determined by thiol labelling with the alkylating agent methyl-maleimide polyethylene glycol [MM(PEG)_24_]. The results showed the complete reduction of PRK, but not FPBase, in the wild-type plants when they were illuminated (Figure 7A).

The analysis of the single mutants showed the severe impairment of the light-dependent reduction of FBPase, PRK, and NADP-MDH in the *ntrc* mutant, compared with the wild type (Figure 7A,B), further confirming the central role of NTRC in the redox regulation of stromal enzymes. Although it was not as affected as the *ntrc* mutant, the *trxm4* mutant showed lower levels of light-dependent reduction of the three enzymes than the *trxm1* mutant (Figure 7A,B). In line with these results, the *trxm1m4* double mutant showed lower levels of light-dependent reductions of PRK and NADP-MDH than the single *trxm1* and *trxm4* mutants, yet it was less affected than the *ntrc* mutant (Figure 7C,D). The mutants with combined deficiencies in NTRC and Trxs *m*1 showed lower levels of light-dependent reduction of the three enzymes, although in the case of FBPase the difference was not statistically significant (Figure 7C,D). Intriguingly, the mutants with combined deficiencies in NTRC and Trx *m*4 showed significantly higher levels of light-dependent reduction of PRK and, to a lesser extent, of FBPase (Figure 7C,D). This finding suggested that the lack of Trx *m*4 might cause the overreduction of other Trxs. To test this possibility, we analysed the light-dependent redox state of Trxs *f.* Interestingly, Trxs *f* were slightly, but consistently, more reduced in the *ntrc-trxm4* and *ntrc-trxm1m4* mutants than in the *ntrc* and *ntrc-trxm1* mutants (Figure S1A–C), suggesting the positive effect of the absence of Trx *m*4 on the light-dependent reduction of Trxs *f*. However, it should be noted that this effect was exclusively observed in the *ntrc* background.

**Figure 8:**
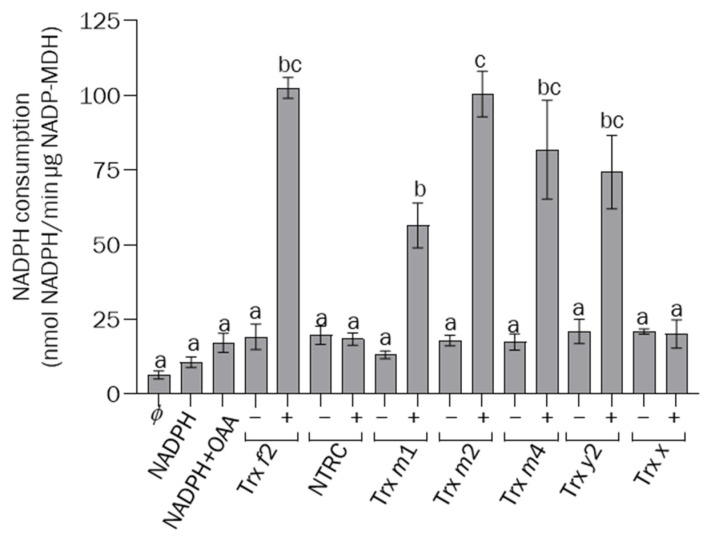
**Analysis of recombinant NADP-MDH activity after treatment with NTRC and chloroplastic Trxs.** NADP-MDH (2 μM) and NTRC/Trxs (1 μM) were mixed in the presence of NADPH/DTT (250/500 μM) for 30 min at RT. Next, they were diluted in buffer containing 300 µM NADPH and 300 µM OAA. The NADP-MDH activity was measured as NADPH consumption, and absorbance was measured at 340 nm for 10 min. *ϕ, s*amples lacking NADP-MDH, substrates and reducing agents were used as negative controls; NADPH and NADPH + OAA, controls including recombinant NADP-MDH and NADPH without/with OAA, respectively. Complete assays with NADP-MDH, substrates, the corresponding Trx or NTRC, without (−) or with (+) reductant, are shown. Determinations were performed three times and mean values ± SE are represented. Letters indicate significant differences (*p* < 0.05) determined by one-way ANOVA, followed by Tukey’s post-test.

Altogether, the light-dependent redox states of FBPase, PRK, and NADP-MDH in the different lines under analysis showed that NTRC activity is needed for the function of type-*m* Trxs; furthermore, these analyses showed the concerted light-dependent redox regulation of CBC and the malate valve by these redox systems. Based on the inability of NTRC to reduce FBPase, as determined by in vitro assays, it was proposed that NTRC regulates FBPase indirectly, via the control of the reducing activity of stromal Trxs rather than through direct interaction with the enzyme [38]. Thus, to establish the mode of regulation of NADP-MDH activity by NTRC, in vitro assays were performed to compare the enzyme activation by different chloroplasts Trxs and NTRC. To this end, N-terminal His-tagged versions of NADP-MDH and Trxs *f*2, *m*1, *m*2, *m*4, *y*2, and *x*, from the Arabidopsis, were expressed in *E. coli*, excluding their corresponding transit peptides, and purified by Nickel chromatography. In agreement with previous results [24,25,26,27,28,29,30,31,32,33,34,35,36,37,38,39,40,41,42,43,44,45,46,47,48,49,50,51,52,53], NADP-MDH was efficiently activated by Trxs *f*2 and *m*2, less efficiently activated by Trxs *m*4, *y*2, and *m*1, and not activated by Trx *x* (Figure 8), revealing the different affinities of the chloroplast Trxs for NADP-MDH. Remarkably, even though the *ntrc* mutant showed the most severe impairment of the redox regulation of NADP-MDH (Figure 7A,B), NTRC was unable to activate NADP-MDH in vitro (Figure 8), which strongly supports the notion that NTRC affects the redox regulation of NADP-MDH indirectly, rather than through the direct activation of the enzyme.

### 3.4. The Lack of 2-Cys Prx B Supresses the Phenotype of ntrc-trxm1m4 Mutant

**Figure 9:**
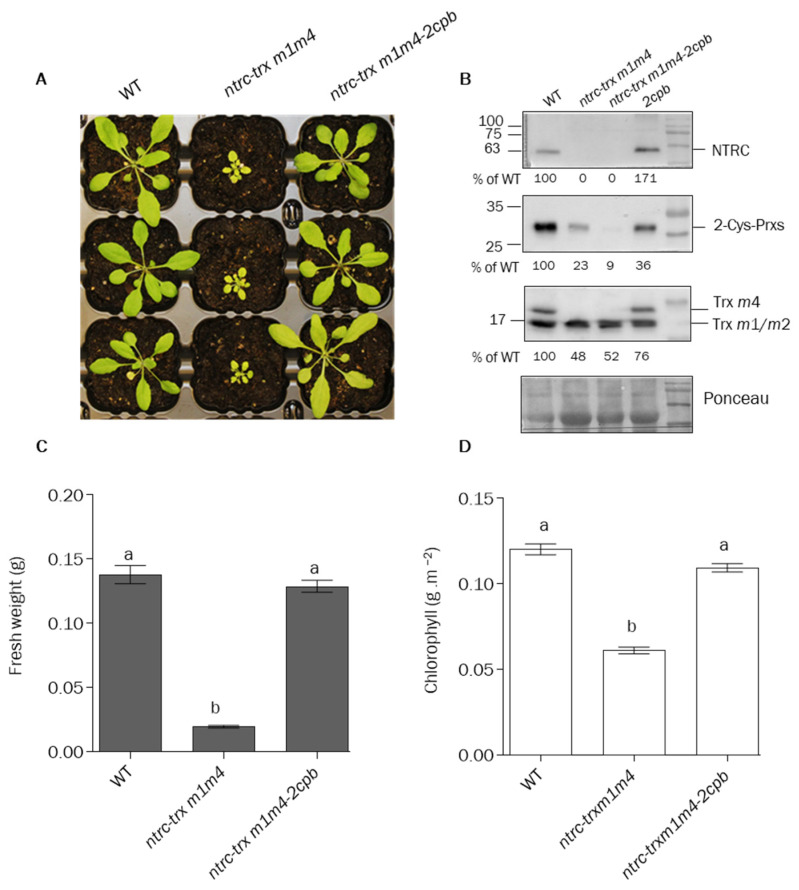
**The phenotype of the *ntrc-trxm1m4* mutant is suppressed by decreased levels of 2-Cys Prxs**. (**A**) Phenotype of wild-type and mutant plants grown under long-day conditions at 120 μE m^−2^ s^−1^ during 21 days. (**B**) The contents of NTRC, 2-Cys Prxs, and/or Trxs *m* in wild−type and mutant lines were analyzed by Western blot. Proteins were extracted from leaves of plants grown under long-day conditions for 3 weeks and subjected to SDS-PAGE (14% polyacrylamide) under reducing conditions, transferred to a nitrocellulose membrane, and probed with an anti-NTRC, anti-2-Cys Prx, or anti-Trx *m* antibody. Ponceau staining was used as a loading control. The *2cpb* was included as a control for the amount of 2-Cys-Prxs. The amount of NTRC, Trxs *m*, and 2-Cys-Prxs in the different lines was quantified and referred to the amount of protein in the Ponceau staining. A representative blot is shown. (**C**) The weights of rosette leaves from at least 12 plants are presented as average values ± SE. (**D**) Chlorophyll content was determined from leaf discs (*n* = 9), and average values ± SE are represented. Letters indicate significant differences (*p* < 0.05), determined by one-way ANOVA, followed by Tukey’s post-test.

The finding that decreased levels of 2-Cys Prxs supress the phenotype of the Arabidopsis *ntrc* mutant [40] uncovered the central role of 2-Cys Prxs in maintaining the reducing capacity of chloroplast Trxs, thereby explaining the ability of NTRC to indirectly affect the redox regulation of Trx targets [9]. Thus, we tested the possibility that the indirect effect of NTRC on NADP-MDH activity might be exerted via the redox balance of 2-Cys Prxs. To this end, the triple *ntrc-trxm1m4* mutant was crossed with the Δ*2cp* mutant, which lacks 2-Cys Prx B and contains low, but detectable levels of 2-Cys Prx A [46]. Despite repeated attempts, no mutant plants simultaneously devoid of Trx *m*4 and 2-Cys Prx A were generated in any of the crosses that were carried out. It was observed that the *Trx m4* and *2-Cys Prx A* genes of Arabidopsis are in the same arm of chromosome 3 and, according to [54], the probability of a recombination event between them is as low as 2.55%, making a *trxm4-2cpa* mutant combination very unlikely. Therefore, we selected the quadruple *ntrc-trxm1m4-2cpb* mutant for further analyses (Figure 9A).

Surprisingly, the levels of 2-Cys Prxs were decreased in the *ntrc-trxm1m4* mutant plants to values lower than those of the *2cpb* mutant plants, whereas the content of these proteins was drastically reduced in the *ntrc-trxm1m4-2cpb* plants (Figure 9B). It was observed that the deficiency in 2-Cys Prx B in the *ntrc-trxm1m4* mutant background resulted in the suppression of the severe growth-inhibition phenotype of the *ntrc-trxm1m4* mutant, as shown by the rosette fresh weight and chlorophyll levels, which were similar to those of the wild type (Figure 9A,C,D). The analysis of the photosynthetic performance of the *ntrc-trxm1m4-2cpb* mutant showed the recovery of the Fv/Fm ratio (Figure S3A) and the partial recoveries of the Y(II) (Figure S3B), Y(NPQ) (Figure S3C), and ETR(II) (Figure S3D), further confirming the suppressor effect of the decreased contents of the 2-Cys Prxs on the impaired photosynthetic performance of the *ntrc-trxm1m4* mutant.

## 4. Discussion

**Figure 10:**
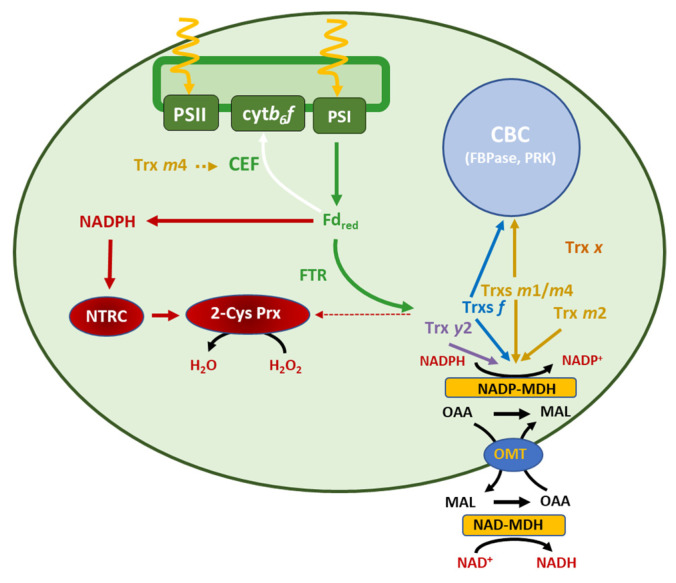
**Role of the Fd–FTR–Trxs and NTRC redox systems in the concerted regulation of Calvin–Benson cycle and the malate valve.** Light-driven electron transport between PSII and PSI generates reduced Fd (Fd_red_), which fuels reducing equivalents for the reduction of Trxs, through the action of Fd–Trx reductase (FTR). The highly abundant *m*- and *f*-type Trxs are involved in the regulation of CBC enzymes (orange and blue arrows), such as fructose-1,6-bisphosphatase (FBPase) [38,40] and phosphoribulokinase (PRK). The NADP-malate dehydrogenase (NADP-MDH), which plays a key role in the maintenance of the balance of the chloroplast NADPH, is reduced by *f*, *m*, and *y*2 Trxs in vitro (orange, blue, and purple arrows). In addition, *m4* Trx regulates cyclic electron flow (CEF, orange dotted arrow) [51]. NTRC, which uses NADPH produced from reduced Fd, plays a central role in the redox regulation of stromal enzymes, and it is needed for the regulatory function of Trxs. NTRC is the main reductant of 2-Cys Prx, a hydrogen-peroxide-scavenging enzyme (red arrow). In the absence of NTRC, reducing power can be drained from the pool of reduced Trxs (red dotted arrow) impairing redox regulation of their targets, revealing the key role of the NTRC-2-Cys-Prx system in the regulation of CBC enzymes and the malate valve.

Given the complexity of the chloroplast-redox network, establishing the hierarchy and specificity of Trxs in the redox homeostasis of the organelle is a challenging issue. Approaches based on biochemical analyses and the generation of Arabidopsis mutants combining the deficiencies in NTRC, different types of Trxs, and 2-Cys Prxs led to the identification of the central role of the NTRC-2-Cys-Prxs redox system in the control of the redox homeostasis of chloroplasts [9]. However, the relationship of NTRC and 2-Cys Prxs with type *m* Trxs, which are the most abundant Trxs in the organelle [32], has been poorly analysed. In this work, we addressed this issue focusing on the concerted redox regulation of the CBC and the malate valve. Our findings and their relationship with previous results are summarized in the scheme shown in Figure 10.

### 4.1. NTRC Is Required for the Trxs-m-Dependent Regulation of CBC and Malate-Valve Enzymes

To address the functional relationship between *m*-type Trxs and NTRC, we generated the double knockout mutant *trxm1m4*, thereby discarding Trx *m*2, because no knockout mutant was available, and Trx *m*3, which has been shown to perform functions that are unrelated to the chloroplast [42]. Previous studies using individual Trx-*m*-deficient mutants suggested that the different isoforms of *m*-type Trxs perform specific functions. This is the case of Trx *m*4, which participates in the regulation of the cyclic electron flow (CEF) [51] (Figure 10, orange dotted arrow), Trx *m*1, which is highly induced under cold stress [55], and Trx *m*2, which has been shown to change its subcellular localisation and regulate a voltage-dependent anion-channel protein in mitochondria [56], as well as performing the reductive activation of FBPase in roots [57]. However, despite these functions of *m*-type Trx isoforms, the single *trxm1* and *trxm4* mutants showed no significant growth-phenotype alterations, at least under the standard long-day conditions used in this study (Figure 2A–C), which is in agreement with previously published results [32,45,58,59]. Thus, the impact of *m*-type Trxs on chloroplast performance and plant growth was analysed in plants with combined deficiencies in these Trxs. Approaches based on the VIGS of the Trxs *m*1, *m*2, and *m*4 resulted in a partial loss of PSII capacity [50]; moreover, RNAi plants with severely decreased levels of Trxs *m* showed PSII photo-inhibition and lethality at the seedling stage [32], indicating the relevant role of type-*m* Trxs in PSII function. However, no mutants devoid of the three major *m*-type Trxs of Arabidopsis have been reported, suggesting that plant viability requires a minimum content of Trxs of the *m* type [32,45]. In line with these results, the double *trxm1m4* mutant, which contains decreased contents of total Trx *m* (Figure 1B), showed growth inhibition and lower contents of chlorophyll (Figure 2A–C). A recent proteomic study showed a closer functional relationship between Trxs *m*1 and *m*4 than with Trx *m*2 [44]; hence, the possibility that the specific lack of Trxs *m*1 and *m*4, rather than a decreased dose of *m*-type Trxs, is responsible for the altered phenotype of the plants cannot be ruled out. Furthermore, the analysis of double and triple Arabidopsis mutants deficient in Trx *m*4, Trx *m*1, and/or Trx *m*2 suggested a relevant role of the Trx *m*4 isoform in plant growth [45].

The NADPH-dependent chloroplast redox system, NTRC, has a deep impact on chloroplast performance [9]. Based on the severe effects of the combined deficiencies in NTRC and Trxs *f* and *x* [36,37,38,41] and, to a lesser extent, NTRC and Trxs *y* [39], the concerted operation of NTRC and the Fd–FTR–Trxs redox systems was proposed. These results suggested that NTRC acts as a central hub modulating the function of all types of chloroplast Trxs (Figure 10, red arrows). A previous report showing the concerted action of NTRC and Trxs of the *m* type on tetrapyrrole biosynthesis [43] suggested that the function of *m*-type Trxs is also modulated by NTRC. Here, we addressed the functional relationship between NTRC and *m*-type Trxs in the coordinated regulation of enzymes in the CBC and the malate valve. The growth-inhibition phenotype shown by the *ntrc-trxm1*, *ntrc-trxm4*, and, more severely, the *ntrc-trxm1m4* mutants (Figure 2A,B) confirms the notion that NTRC is required for the function of Trxs *m*1 and *m*4. Moreover, the significant reduction in the contents of chlorophyll (Figure 2A,C), agreed with the impairment of tetrapyrrole biosynthesis in plants with silenced Trxs *m* in the *ntrc* background [43], and confirmed the findings in previous reports, which showed the participation of NTRC and Trxs *m* in the regulation of chlorophyll biosynthesis [22,23].

The altered phenotype caused by the lack of NTRC and the Trxs *m*1 and *m*4 was observed in all of the parameters analysed, i.e., the *ntrc-trxm1* and *ntrc-trxm4* mutants had impaired photosynthesis efficiency, as shown by their higher levels of NPQ (Figure 4A), as well as lower levels of Y(II) (Figure 4B) and ETR(II) (Figure 5) than the *ntrc* mutant, an effect that was aggravated by the combined deficiencies in NTRC and the Trxs *m*1 and *m*4. Additionally, the combined deficiency in NTRC and the Trxs *m*1 and *m*4 resulted in alterations in the chloroplast structure, which were also more severe in the triple *ntrc-trxm1m4* mutant (Figure 6). These results further support the notion that NTRC activity is needed for the regulatory function of Trxs *m* and emphasize the previously proposed role of these Trxs in PSII biogenesis [50]. In this regard, the *trxm1m4* mutant showed the lowest Fv/Fm ratio of the lines under analysis (Figure 3), which was in contrast with the lack of effect observed in mutants lacking Trxs *f* or Trx *x* [33,38]. These results revealed the specific role of Trx *m*1 and *m*4 in PSII stability, in agreement with previous results showing the participation of Trxs *m* in the protection of the photosynthetic apparatus [60] and, specifically, of Trx *m*4 in the regulation of PSII maximum quantum yield [45]. Remarkably, the similarity in the Fv/Fm values in the *trxm1m4* and *ntrc-trxm1m4* mutants (Figure 3) indicated that this function of Trxs *m* is not affected by NTRC.

A key function of the chloroplast-redox regulatory network is the optimization of the balance of reducing equivalents in the form of NADPH, which is used for assimilatory purposes or exported from the organelle via the malate valve in response to changes in light intensity [52] (Figure 10, black arrows). Thus, an important aspect of the control of the chloroplast NADP^+^/NADPH ratio is the concerted regulation of CBC enzymes and NADP-MDH. The regulation of NADP-MDH is necessary for the maintenance of chloroplast-NADPH poise, especially under short-day and fluctuating-light conditions, as revealed by the analysis of mutant plants containing NADP-MDH whose activity was independent of light [61]. The additive effect of the lack of NTRC and the Trxs *m*1 and *m*4 on plant growth (Figure 2A–C), photosynthetic performance (Figure 4A,B and Figure 5), and chloroplast stability (Figure 6) lends further support to the functional relationship between NTRC and the Trxs *m*1 and *m*4 in the coordinated regulation of the CBC and the malate valve (Figure 10, orange arrows). The lack of Trx *m*1, *m*4, or both caused deficient light-dependent reductions in NADP-MDH (Figure 7A–D), in agreement with the lower activation of NADP-MDH in mutant plants lacking Trxs *m*1 and *m*2 [59]. In contrast, the light-dependent reduction of the CBC enzyme PRK was more affected than FBPase in response to the lack of Trxs *m*1 and *m*4 (Figure 7A–D). In this regard, it should be mentioned that PRK is reduced in vitro by the Trxs *f*1, *m*1, and *m*2, but not by Trxs *m*3 and *m*4 [62]. Therefore, Trxs of the *m* type show different regulatory effects on different enzymes in the CBC. Remarkably, both the light-dependent reduction of the malate-valve enzyme NADP-MDH and the CBC enzymes FBPase and PRK were more impaired in the *ntrc* mutant (Figure 7A–D), which confirmed the central function of NTRC in the coordinated regulation of the two pathways. Surprisingly, the level of light-dependent reduction of PRK was increased in the mutants with a combined lack of NTRC and Trx *m*4 (Figure 7C,D). The higher level of reduction in Trx *f* in these mutants (Figure S1A–C) suggests that the absence of Trx *m*4, which is an abundant chloroplast Trx [32], alters the redox state of the other chloroplast Trxs, causing differences between the levels of light-dependent reduction of PRK, FBPase, and NADP-MDH (Figure 7C,D).

### 4.2. The Function of NTRC and m-Type Trxs in Chloroplast Performance Depends on the Levels of 2-Cys Prxs

Our results, showing the impairment of the light-dependent reduction of NADP-MDH in the *ntrc* mutant, agreed with previous results indicating the participation of NTRC in the regulation of NADP-MDH [59]. Moreover, plants devoid of NADP-MDH show increased levels of NTRC, suggesting that NTRC acts as a compensatory mechanism to prevent oxidative stress when the malate valve is not operative [63], further suggesting the functional relationship between NTRC and NADP-MDH. However, while Trxs *f*1 and *m*2 reduced NADP-MDH in vitro with different efficiencies, NTRC failed to reduce the enzyme [24]. We extended this analysis to other Trx isoforms, showing similar efficiency in the reduction in NADP-MDH by Trxs *m*2 and *f*2 and, to a lesser extent, by Trxs *m*4 and *y*2, whereas Trx *x* failed to reduce the enzyme in in vitro assays (Figure 8). However, most importantly, our results further confirmed the failure of NTRC to reduce NADP-MDH (Figure 8), in agreement with previous results [24]. Therefore, NTRC may exert its regulatory effect on NADP-MDH indirectly, via the modulation of the redox balance of 2-Cys Prxs, as was previously proposed for the regulation of CBC enzymes [38], by minimizing the drainage of reducing equivalents from the pool of Trxs (Figure 10, dotted red arrow). This possibility was addressed in this study by the generation of the quadruple mutant *ntrc-trxm1m4-2cpb*. Interestingly, the *ntrc-trxm1m4* plants showed reduced contents of 2-Cys Prxs compared to the wild-type plants, with levels below those of the *2cpb* mutant plants. Decreases in 2-Cys-Prx protein levels, reaching 75% of the protein content in the wild-type plants, and in the expression of both *2cpa* and 2*cpb*, were previously reported in *ntrc* mutant plants, and were further decreased in the *ntrc-trxx* double mutant [41]. Unfortunately, the proximity of the *Trx m4* and *2-Cys Prx A* genes in the same arm of chromosome 3 prevented us from obtaining mutant plants lacking 2-Cys Prx A in the *trxm4* background. However, the drastic decrease in the 2-Cys Prx protein in the *ntrc-trxm1m4-2cpb* plants was sufficient to suppress the *ntrc-trxm1m4* phenotype (Figure 9A–D). This suppressor effect of the growth-inhibition phenotype was robust and extended to all of the photosynthetic parameters analysed in this study, such as Fv/Fm (Figure S3A), Y(II) (Figure S3B), Y(NPQ) (Figure S3C), and ETR(II) (Figure S3D). These results suggest that the redox imbalance of 2-Cys Prxs in the absence of NTRC provokes the drainage of electrons from the Trxs *m*1 and *m*4, as was previously shown for the Trxs *f* and *x* [40,41].

In summary, as depicted in the scheme shown in Figure 10, the severe phenotypic effects of the simultaneous deficiency in NTRC and the Trxs *m*1 and *m*4 support the relevant role of these Trxs in photosynthetic performance and plant growth. Both NTRC and Trxs *m*1 and *m*4 participate in the concerted regulation of CBC and malate-valve enzymes, ensuring the adequate balance of the chloroplast-redox state, which allows the modulation of assimilatory pathways and the extrusion of reducing equivalents in response to variations in light intensity. The almost-wild-type phenotype of the *ntrc-trxm1m4-2cpb* mutant shows the suppressor effect of highly decreased contents of 2-Cys Prxs, indicating the central function of the NTRC-2-Cys-Prxs redox couple in the modulation of the reducing activity of different chloroplast Trxs.

## Data Availability

The data presented in this study are available on request from the corresponding author.

## Supplementary Materials

The following supporting information can be downloaded at: https://www.mdpi.com/article/10.3390/antiox12051041/s1. Table S1: Primers used for genotyping the mutant lines analysed in this work; Table S2: Primers used for gene-expression analysis by RT-qPCR; Table S3: Oligonucleotides used for protein cloning in pQE30 vector; Figure S1: Analysis of the in vivo redox state of Trxs *f* in plants lacking NTRC and/or Trxs *m*; Figure S2: Purification of N-terminal tagged recombinant proteins; Figure S3: Photosynthetic performance is restored in *ntrc-trxm1m4* by decreased levels of 2-Cys Prxs.
