# Supplementary material for: The Functional Relationship between NADPH Thioredoxin Reductase C, 2-Cys Peroxiredoxins, and m-Type Thioredoxins in the Regulation of Calvin–Benson Cycle and Malate-Valve Enzymes in Arabidopsis
Source: Antioxidants (Basel). 2023 May 3;12(5):1041. doi: 10.3390/antiox12051041 (PMC10215429; doi:10.3390/antiox12051041)
Supplement: Supplementary file 1 [file antioxidants-12-01041-s001.zip › antioxidants-2344082-supplementary.pdf]

Table S1. Primers used for genotyping the mutant lines analysed in this work.

Gene	Locus	Primer name	Sequence (5'-3')
NTRC	At2g41680	NTRC-Fsalk	TCACCAACATGTGGCCC
		NTRC-Rsalk	TTCTTCATCTTCACACCCGA
Trx m1	At1g03680	5Atm1	GGGCTTTAATGAAACTGCGG
		3Atm1	ACGGGAAGTACACGTGTAAG
Trx m2	At4g03520	5Atm2	CTTTGATTCGTGCTCGTTGG
		3Atm2	ATCTGAGTAGGTACCCAACC
Trx m4	At3g15360	5Atm4-2	GAGCCCAAATACAGCAAACC
		3Atm4-2	GAAGAAGACAAAGAGGACCC
2cpa	At3g11630	2cpaGK-RP	CTTCCACTGGTTGGAAACAAG
		2cpaGK-LP	AATGCCTGCAACATTGAAAAC
2cpb	At5g06290	2cpb-F1	CCACCTGAACCAAGAAAG
		2cpb-R1	CCTGCAAGACAACATCAC
**SALK T-DNA**	T-DNA	Lb1.3	ATTTTGCCGATTTCGGAAC

Table S2. Primers used for gene expression analysis by RT-qPCR.

Gene	Locus	Primer name	Sequence (5'-3')
NTRC	At2g41680	q-NTRC-F1	TGAAGATGAAGAAAGAGTACCGAG
		q-NTRC-R1	GGTGTCCTCATTTATTGGCCT
Trx m1	At1g03680	AtTRXM1-qfor	AATTCTAGGGTTTCCCGATTACG
		AtTRXM1-qrev	GAGTCCCATGTTGAATCGTTGA
Trx m2	At4g03520	AtTRXM2-qfor	TCTCCGGCTTCGTTGACC
		AtTRXM2-qrev	GAGCTTCACAGACGACGGCT
Trx m4	At3g15360	AtTRXM4-qfor	AATCGCTCGCGGTGAC
		AtTRXM4-qrev	GATTTGGTACTTCGACGGCG
Trx f1	At3g02730	TRX f1 qFw	CGATGATCTGGTTGCAGCG
		TRX f1 qRv	CTGGTTCATCCGGAAGCAG
Trx f2	At5g16400	TRX f2 qFw	TGTAACCAAGACAACAAGCCA
		TRX f2 qRv	CGGTCACTTCCTTTACTACCT
Actin	At3g18780	Act-qFor	GCACTTGCACCAAGCAGCAT
		Act-qRev	CCTTTCAGGTGGTGCAACGAC
Ubq10	At4g05320	UBQ10-qfor	GGCCTTGTATAATCCCTGATGAATAAG
		UBQ10-qrev	AAAGAGATAACAGGAACGGAAACATAAGT

Table S3: Oligonucleotides used for protein cloning in pQE30 vector. Restriction sites are underlined.

Gene	Primer name	Sequence (5'-3')
Trx m1	Attrxm1-BamHIF	CAGGATCCCTATCTTCACTCTCGAAGAATTCT
	Attrxm1-HindIIIR	CAAAGCTTTTACAAGAATTTGTTGATGCTGG
Trx m2	Attrxm2-BamHIF	CCGGATCCTGTGAAGCTCAGGAAACTACTAC
	Attrxm2-HindIIIR	CAAAGCTTTCATGGCAAGAACTTGTCG
NADP-MDH	NADPMDH-BamHIF	CAGGATCCTGCTCCGTTTCTCAAAATAG
	NADPMDH-HindIIIR	CAAAGCTTTCAAACTTCCCCAGGAAG

Fig. S1

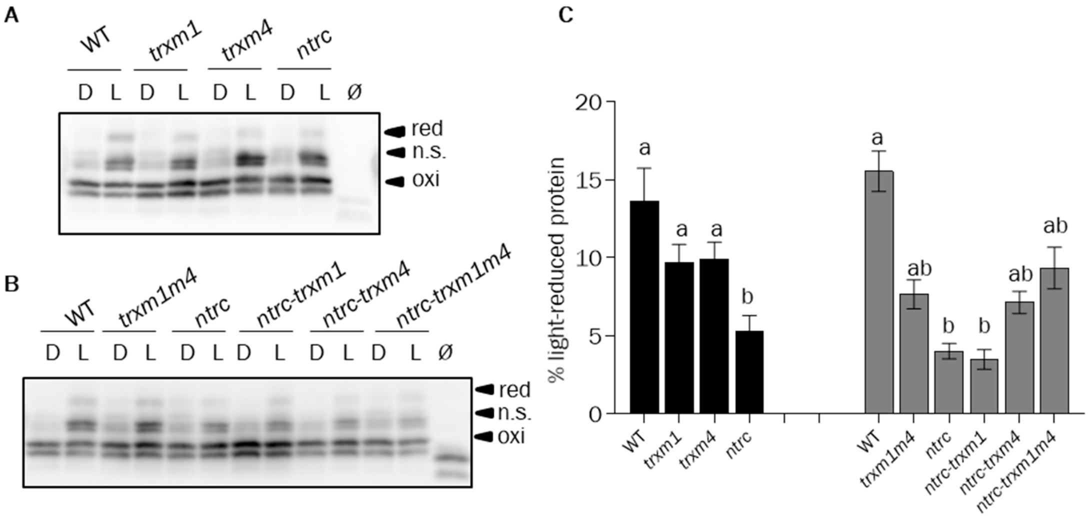
A
WT
trxm1
trxm4
ntrc
D L D L D L D L Ø
red
n.s.
oxi
B
WT
trxm1m4
ntrc
ntrc-trxm1
ntrc-trxm4
ntrc-trxm1m4
D L D L D L D L D L D L Ø
red
n.s.
oxi
C
% light-reduced protein
0
5
10
15
20
a
a
a
b
a
ab
b
b
ab
ab
WT
trxm1
trxm4
ntrc
WT
trxm1m4
ntrc
ntrc-trxm1
ntrc-trxm4
ntrc-trxm1m4

Fig. S2

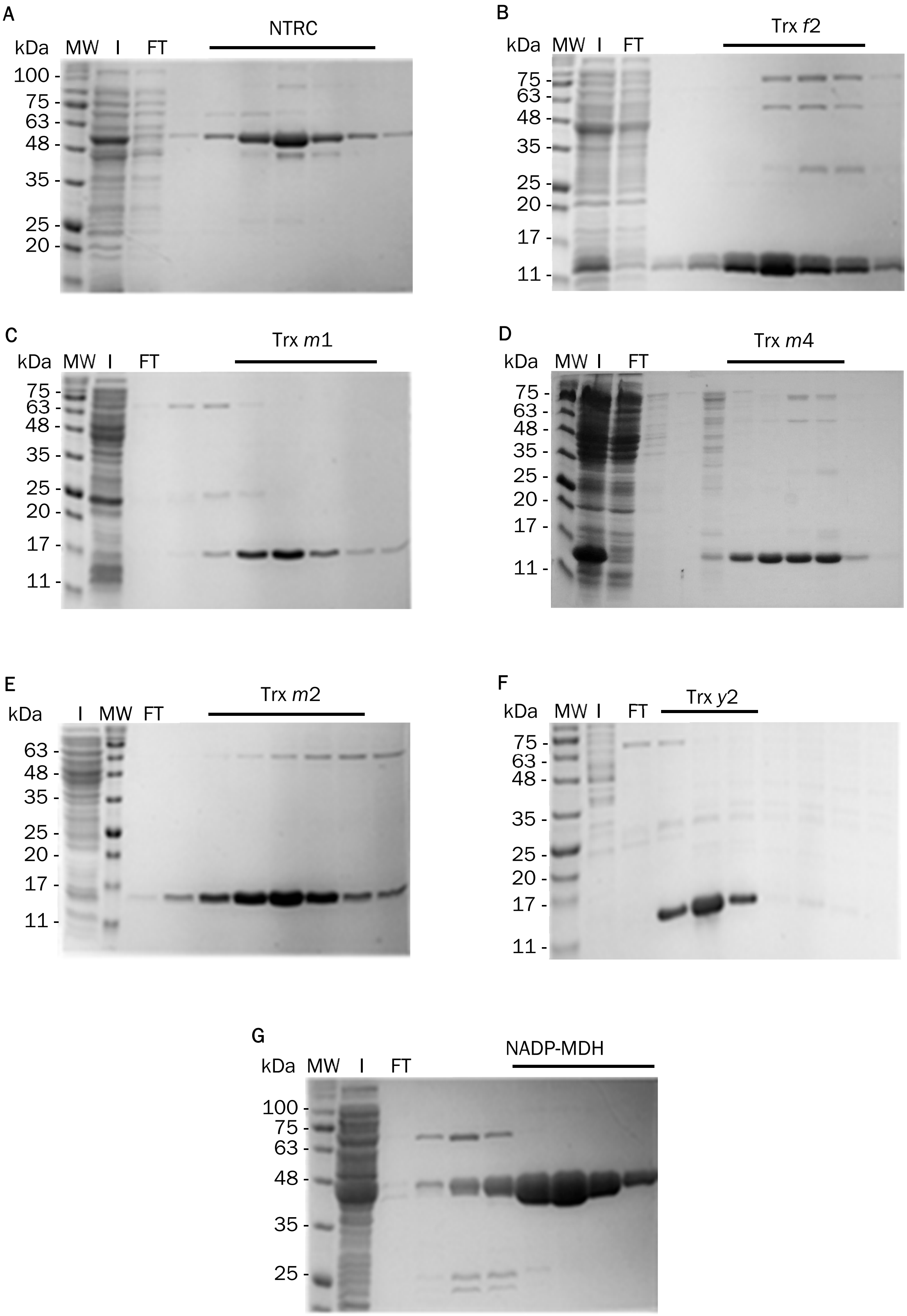
A
kDa MW I FT
NTRC
100
75
63
48
35
25
20
B
kDa MW I FT
Trx f2
75
63
48
35
25
20
17
11
C
kDa MW I FT
Trx m1
75
63
48
35
25
20
17
11
D
kDa MW I FT
Trx m4
75
63
48
35
25
20
17
11
E
kDa I MW FT
Trx m2
63
48
35
25
20
17
11
F
kDa MW I FT
Trx y2
75
63
48
35
25
20
17
11
G
kDa MW I FT
NADP-MDH
100
75
63
48
35
25

Fig. S3

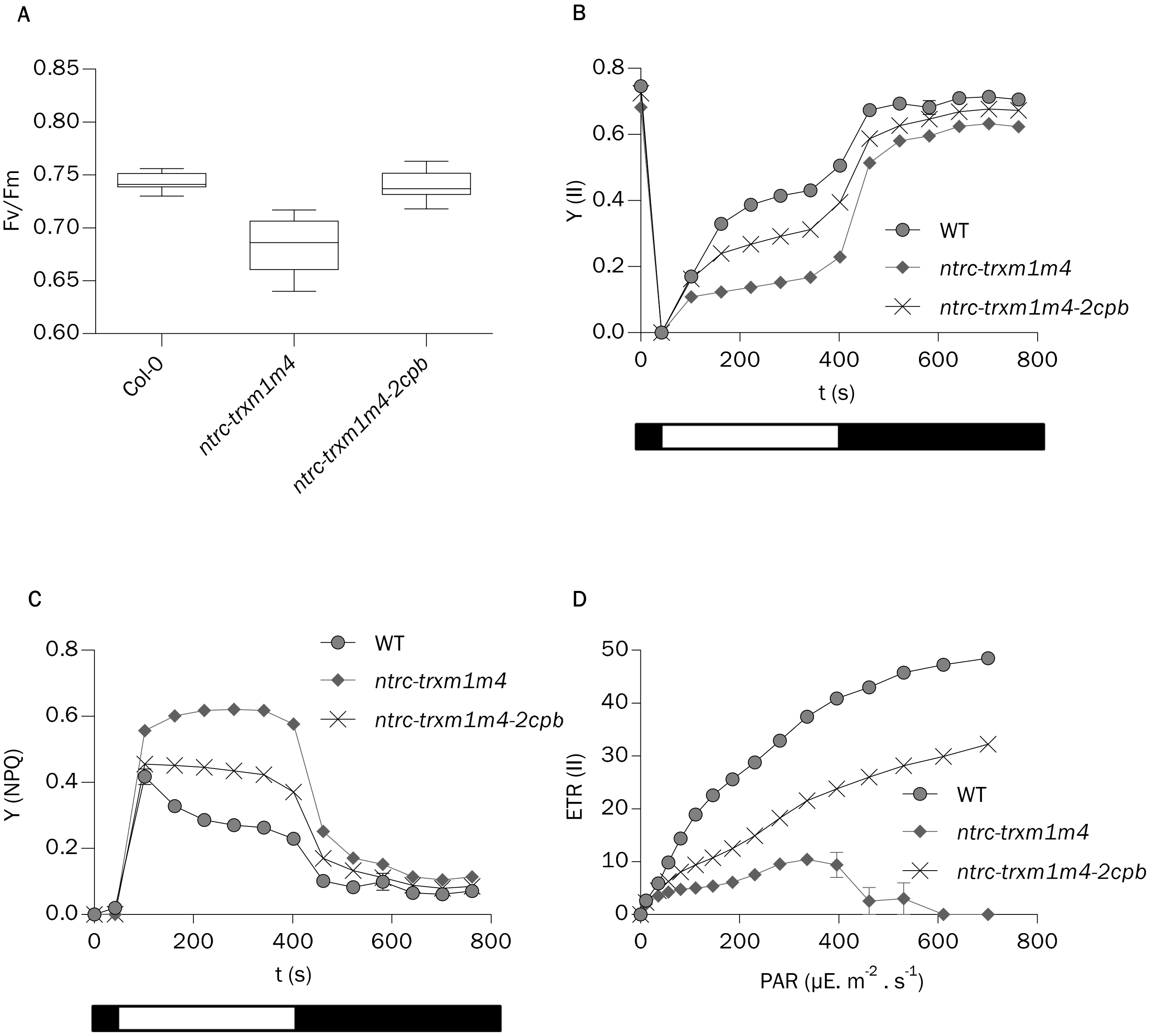

A
Fv/Fm
0.85
0.80
0.75
0.70
0.65
0.60
Col-0
ntrc-trxm1m4
ntrc-trxm1m4-2cpb
B
Y (II)
WT
ntrc-trxm1m4
ntrc-trxm1m4-2cpb
t (s)
C
Y (NPQ)
WT
ntrc-trxm1m4
ntrc-trxm1m4-2cpb
t (s)
D
ETR (II)
WT
ntrc-trxm1m4
ntrc-trxm1m4-2cpb
PAR (µE. m-2 . s-1)

Figure legends:

Figure S1. Analysis of the *in vivo* redox state of Trxs *f* in plants lacking NTRC and/or Trxs *m*. WT and mutant plants were grown under long-day conditions for 22 days at a light intensity of 120 $\mu E \cdot m^{-2} s^{-1}$. Plants were incubated in darkness for 5 hours and dark-adapted samples were collected (D), then light was switched on and samples were taken after 30 min at a light intensity of 480 $\mu E \cdot m^{-2} s^{-1}$ (L). The *in vivo* redox state of Trxs *f* was determined in WT, single mutant plants (A, C), as well as multiple mutants (B, C). Proteins were extracted in the presence of 10% TCA to preserve the thiol redox state. Protein thiols were alkylated with 10 mM $MM(PEG)_{24}$ prior to electrophoresis in a 9.5% polyacrylamide gel (14% for Trxs *f)* under reducing conditions, transferred to a nitrocellulose membrane, and probed with the indicated antibodies. red, reduced; ox, oxidized; n.s., non-specific band. (C) The corresponding band intensities were quantified (GelAnalyzer), and the percentage of reduction is the ratio between the reduced form and the sum of reduced and oxidized forms for each protein. Each value is the mean of three independent experiments ± SE. Letters indicate significant differences ($P < 0.05$) determined by one-way ANOVA followed by Tukey's post-test.

Figure S2: Protein purification*.* Recombinant NTRC (A), Trx *f*2 (B), Trx *m*1 (C), Trx *m*4 (D), Trx *m*2 (E), Trx *y*2 (F) and NADP-MDH (G) were purified from the corresponding strains by Ni^{2+} affinity chromatography and the resulting fractions analysed by SDS-PAGE (9 to 15% acrylamide) under reducing conditions. MW, molecular weight marker; I, column input; FT, column flow through. Black bars indicate the fractions used for *in vitro* assays.

Figure S3. Photosynthetic performance is restored in *ntrc-trxm1m4* plants by decreased levels of 2-Cys Prxs. Photosynthetic parameters were measured on whole rosettes of the wild-type and mutant plants, as indicated, grown under long-day conditions for 21 days (A) Fv/Fm was determined in dark adapted plants. Box plots indicating the Fv/Fm value are shown. In each case, the median (segment inside rectangle), upper and lower quartiles (boxes), and minimum and maximum values (whiskers) are indicated. (B) Quantum yields of photosystem II photochemistry, Y(II), and (C) non-photochemical quenching, Y(NPQ). Each value is the average of four determinations, and mean values ±SE are represented. White and black blocks indicate periods of illumination with actinic light (81 $\mu E\ m^{-2}\ s^{-1}$) and darkness, respectively. (D) Relative ETRs of PSII, ETR(II), were determined during stepwise increase of photosynthetically active radiation (PAR) in 3 replicates and each data point is the mean ± SE.
